# The In-Hospital Code Stroke: A Look Back and the Road Ahead

**DOI:** 10.1177/19418744241298035

**Published:** 2024-10-29

**Authors:** Andrea M Kuczynski, W David Freeman, Lesia H Mooney, Josephine F Huang, Andrew M Demchuk, Houman Khosravani

**Affiliations:** 1 Division of Neurology, Department of Medicine, 7938 University of Toronto, Toronto, ON, Canada; 2 Hurvitz Brain Sciences Program, Sunnybrook Health Sciences Centre, 7938 University of Toronto, Toronto, ON, Canada; 3 Department of Neurologic Surgery, Neurology, and Critical Care, Mayo Clinic, Jacksonville, FL, USA; 4 Division of Neurology, Department of Medicine, Cumming School of Medicine, 2129 University of Calgary, Calgary, AB, Canada

**Keywords:** acute stroke, ischemic stroke, hemorrhagic stroke, inpatients, quality improvement

## Abstract

With increased patient volumes and complexity, stroke occurrence in hospitalized patients has become relatively more common. The process of activating a code stroke in-hospital differs in many institutions. An emergency team-based response to inpatient acute code stroke is warranted, with many protocols modeled similarly to the cardiac arrest response. However, several studies have demonstrated delays in recognition and management of acute stroke in-hospital as compared to those arriving directly to the emergency department (ED). Furthermore, there are several shared challenges with code stroke resuscitation in the emergency department and the ward, which include the assembly of ad hoc teams and requirement of access to urgent imaging. Delays in activating in-hospital code stroke contributes to increased morbidity, mortality, prolonged hospitalization, and associated health care costs. In the following commentary, we discuss the current landscape of acute in-hospital code stroke protocols, review the differences in neurologic outcomes between inpatient vs ED/out-of-hospital code stroke patients, and propose future directions for in-hospital code stroke paradigms for improved patient outcomes and quality of care.

## Introduction

In-hospital acute strokes account for 7% to 15% of acute cerebrovascular events experienced by inpatients.^1-3^ Studies have identified older age^4,5^ and a higher prevalence of comorbid conditions including atrial fibrillation,^4,6-9^ elevated or unstable blood pressure, cerebrovascular stenosis, and remote or recent myocardial infarction,^9^ as risk factors for stroke in inpatients compared to those presenting from out-of-hospital.^5,10,11^ Additionally, National Institutes of Health Stroke Scale (NIHSS) scores,^4,10^ Canadian Neurologic Scale (CNS) scores,^5^ or modified Rankin Scale (mRS) scores^12^ have typically been higher in patients in-hospital, conferring a greater initial disability, which is likely attributed to older age and increased disease burden. There are several unique causes of in-hospital stroke including perioperative stroke, most commonly occurring after cardiovascular procedures, angiography,^4,5,7^ and unsuccessful cardioversion. Inpatient procedures also often necessitate interruption of anticoagulation which further increases stroke risk. This risk profile for stroke is more often attributed to embolic causes of ischemia compared to out-of-hospital stroke patients.^3,7^ It has further been postulated that atrial fibrillation is associated with greater risks of other heart disease,^13^ infection, and dehydration, conferring an even greater risk of embolic events during hospitalization.^4,14^ A recent statement from the American Heart Association has summarized the current limitations and posed future directions of in-hospital acute strokes.^15^ In the following commentary, we will discuss barriers in in-hospital stroke and areas for improvement.

## Methods

We conducted a literature review using a comprehensive Medline (PubMed) search to identify publications related to in-hospital acute stroke protocols. The following search terms were used: “in-hospital stroke”, “inpatient” (permutations of these), and terms such as “code stroke”, “stroke alert”. We also conducted an ancillary search using GoogleScholar. Searches were not limited by date. Inclusion criteria included manuscripts published in the English language, on adults with ischemic stroke in the in-hospital setting, and reporting on clinical outcomes. Titles and abstracts of all retrieved publications were screened for relevance, and full-text manuscripts that aligned with the focus of our review were reviewed.

## Results

Our search resulted in 35 articles, and 28 manuscripts met our inclusion criteria. Our review revealed significant variability in protocols and outcomes among studies, reflecting heterogeneity in hospital resources and patient populations. This heterogeneity poses challenges in drawing definitive conclusions and underscores the need for standardized protocols and outcome measures. Additionally, most studies had small sample sizes and lacked long-term follow-up, limiting the generalizability of their findings. None of these studies compared length of hospital stay pre- and post-implementation to ascertain whether timely investigation and treatment of stroke affected discharge. In the future, neurologic and functional outcome measures should be consistent among cohort studies comparing inpatient vs out-of-hospital stroke.

## The Current Landscape and the Clinical Challenge

### Inpatients Face Increased Risk of Stroke and Worse Outcomes

In the setting of an aging population and overall higher acuity of patients, increased incident risk of stroke for inpatients confers a higher risk of worse outcomes. This is in part due to comorbidities and hospitalization but also due to greater delays in their care (Table 1). One such delay is the lack of recognition of the signs and symptoms of a stroke in some hospitalized patients, with possible causes including less knowledge of stroke symptoms by members of the health care team, and delirium and/or medication being attributed to a change in the patient’s level of consciousness or cooperativity. The timing of when a patient is last seen well is often not ascertained as readily in the context of in-hospital code strokes.^16^ Furthermore, members of the health care team may choose to monitor the patient more closely to assess for further symptom development rather than performing neuroimaging or activating a code stroke. This differs from out-of-hospital strokes where pre-hospital notification reduces median door-to-neuroimaging times.^17^ These delays not only impact the amount of neuronal loss associated with stroke and neurologic outcomes, but also the eligibility for intravenous thrombolytic therapy if symptom onset falls outside of the traditional 4.5-hour window.^18^ Ideally, door-to-needle time for thrombolytic therapy should be < 30 minutes, whereas a door-to-reperfusion time for endovascular therapy should be < 120 minutes.^19,20^ Despite being within the 4.5-hour window for thrombolysis, a study by Saltman et al. (2015) observed lower rates of thrombolysis (12% vs 19% in outpatients, *P* < 0.001) and longer door-to-needle times for thrombolysis (median 2.0 hours vs 1.2 hours, *P* < 0.001) in in-hospital stroke.^5^ The rates of thrombolysis are lower in patients already admitted to hospital experiencing an acute ischemic stroke often due to exclusionary criteria, such as recent surgeries, active bleeding, or anticoagulant use. Furthermore, there is restriction on the role of thrombolysis for more minor stroke cases, and more severe cases with contraindications in the inpatient setting further drives the utility of endovascular thrombectomy (EVT) for this group. We postulate that relative to EVT, thrombolytic rates may be lower in certain instances and yet the proportion of patients exhibiting delays remains due to process factors. However, delays in recognition and treatment can still hinder timely access to (EVT), highlighting the need for improved processes regardless of treatment modality. Several studies have revealed major differences in time metrics between in-hospital vs out-of-hospital stroke. Garcia-Santibanez et al (2015) reported delays in symptom onset to neuroimaging (32.3 minutes vs out-of-hospital 14.9 minutes, *P* < 0.001) and tissue-type plasminogen activator (tPA) administration (80.3 minutes vs out-of-hospital 59.3 minutes, *P* = 0.01), with the greatest delay noted as the time to obtain neuroimaging.^21^ Similarly, Masjuan et al. (2008) noted increased duration between door-to-neuroimaging time (39.5 ± 18.7 minutes vs out-of-hospital 22.6 ± 19.7 minutes, *P* < 0.0001) and neuroimaging-to-tPA time (92.0 ± 26.1 minutes vs out-of-hospital 65.4 ± 25.8 minutes, *P* < 0.0001) in in-hospital strokes.^8^

**Table 1.** Comparison of In-Hospital Vs Out-Of-Hospital Acute Stroke Characteristics.

	Inpatients	Outpatients
Age	Older	Younger
Comorbidities	Greater prevalence of comorbidities including cardiovascular risk factors (eg, atrial fibrillation, hypertension, coronary artery disease) and higher likelihood of embolic etiologies of stroke, higher likelihood of being more unstable due to other medical issues necessitating hospitalization	Fewer comorbidities
Anticoagulation	More often have therapeutic anticoagulation held in the setting of in-hospital procedures and surgeries	More often anticoagulated for vascular risk factors, if compliant with medications at home
Time from symptom onset to activation of stroke team	Greater delays in recognition of stroke symptoms and/or activation of a stroke response	Often fewer delays in recognition of symptoms and faster assessment
Time from onset to imaging	Delays due to late recognition, mobilization, often sicker patient	Less delays
Time from onset to intervention (thrombolysis and/or EVT)	Longer times	Shorter times
Rates of thrombolysis	Lower	Higher
Documentation and stroke care	Often more omissions in documentation, investigations missing (eg, holter monitor, swallowing assessments)	More streamlined approach to documentation, investigations and allied health assessments
Clinical outcomes	Rates of discharge and independence often lower due to comorbidities and complications	Higher likelihood of discharge and functional independence

Part of these delays in obtaining neuroimaging in in-hospital stroke has been attributed to longer distances to the scanner compared to those for patients initially arriving at the emergency department. An additional delay in in-hospital stroke has been observed with delayed notification and evaluation by stroke teams.^22^ In further comparison of in-hospital and out-of-hospital code stroke patients, those with in-hospital stroke tended to have less neuroimaging, Holter monitoring, care on an acute stroke ward, and swallowing assessments.^5^ One study by Farooq et al. (2007) noted that in-hospital stroke patients were less likely to have cerebrovascular investigations by any method (55.2% vs 75.6% for out-of-hospital stroke, *P* < 0.01), and more details missing from their documentation compared to out-of-hospital strokes.^7^ Similarly, Aly et al. (2000) also noted reduced documentation in in-hospital stroke patients.^9^ These delays to, and reduced rates of investigation and treatment likely contribute to in-hospital stroke patients having higher rates of mortality and greater mRS scores at discharge.^4,5,7,9^ The likelihood of discharge home is further reduced in patients who experience stroke during their hospitalization, and length of hospital stay tends to be longer in patients with in-hospital stroke.^5,7^ This in turn causes indirect costs for individuals with stroke who may experience delays in returning to work or incur the costs of rehabilitation.

### Standard In-hospital Code Stroke Protocols

Another barrier to the in-hospital code stroke is the lack of standardized protocols. To our knowledge, seven studies have compared outcomes pre- and post-implementation of an in-hospital acute code stroke protocol.^2,23-28^ These studies utilized education of key stakeholders and responders including nursing units, physicians, telecommunication operators and imaging technologists, as well as feedback mechanisms to refine their stroke protocols. The details of their protocols are summarized in Supplemental Table 1. Common screening tools were variable and included the FAST mnemonic (Facial drooping, Arm numbness or weakness, Slurred speech, and Recognition time within 6 hours of last known well),^24^ FAST-DAN (FAST, Deviation of eyes, Aphasia, Neglect) menmonic,^27^ the Cincinnati Prehospital Stroke Scale (arm drift, facial droop, or speech disorder),^26^ and the RACE mnemonic (facial palsy, arm motor impairment, leg motor impairment, head and gaze deviation).^23^ Other studies specifically targeted any new onset of any of the following symptoms as an indication to activate an in-hospital code stroke: unilateral arm and/or leg weakness, difficulty speaking, and/or facial weakness.^2^

In general, these cohort studies observed significantly reduced time intervals from symptom recognition to notification, symptom onset to neuroimaging, and symptom recognition to intravenous thrombolysis (Supplemental Table 1). Following stroke protocol implementation, Kassardjian et al (2017) reported a larger proportion of milder strokes being identified (78.1% vs 48.1%, *P* = 0.001),^2^ and Drogemeuller et al (2020) similarly noted increased incidence of stroke of 6%, as well as a stroke mimic rate of 68%, though their study did not compare mimic rates pre-implementation to allow for further comparison.^24^ This differed from Manners et al. (2019) who noted increased rates of stroke mimics post-implementation.^23^ Two studies^26,27^ that evaluated long-term outcomes (i.e., mRS and discharge location) found no differences following implementation of the in-hospital stroke paradigm; however, Kawano et al. (2021) noted improved clinical outcomes at discharge and at 3-months in patients who were directly examined by a neurologist before any investigations in the in-hospital code stroke protocol.^27^ Additionally, Manners et al (2019) noted improvements in documentation following post-protocol implementation,^23^ and one retrospective review also identified an increased likelihood of last known well time documentation following acute stroke education.^29^

While clinically relevant and demonstrating significant improvements in the timing of investigations and treatment from symptom onset, these studies were not without limitations. In terms of personnel education and stroke team members, the included individuals varied across the studies. Yoo et al. (2016) focused on the cardiology and cardiovascular surgery nurses and physicians and utilized the emergency department personnel and imaging.^25^ In the study by Drogemeuller et al (2020), the inpatient response team included a neurologist, the patient’s primary nurse, a critical care physician, stroke coordinator, radiologist, pharmacist, respiratory therapist, and security services personnel.^24^ Koge et al. (2017) included the patient’s primary physician, either a stroke nurse coordinator or on-call neurologist during off-hours, and emergency department nurse.^26^ In the study conducted by Manners et al. (2019), a neurologist, critical care nurse and physician, radiologist, and the patient’s primary physician were included in the code team.^23^ Kassardjian et al. (2017) included the patient’s primary nurse and physician, and a neurologist in the acute team.^2^ Finally, Kawano et al. (2021) included an acute stroke team comprised of the primary care nurse and physician and stroke neurologist.^27^ It is possible that other personnel were included in the acute response teams, but further details were not included.

### Members of the Team and Nursing Empowerment

Most in-hospital stroke teams have consisted of 2 components: A) the patient’s most responsible team including their physician (often a non-neurology physician), primary nurse, and the unit charge nurse, and B) the converging stroke response team comprised of a neurologist or physician member of the neurology team, stroke coordinator, and a specialized nurse. Facilitated by telecommunications operators, these teams converge at the patient’s bedside for handover of pertinent clinical information including past medical history and reason for hospitalization, hospital medication use such as antiplatelets or anticoagulants, clinical signs or symptoms prompting activation of the code, and time of last known well. In some cases, respiratory therapists have also been involved in the converging stroke team, given often greater respiratory instability of inpatients. The involvement of a code stroke coordinator or a specialized stroke nurse able to perform the NIHSS and prepare the patient for stroke treatment (i.e., intravenous thrombolysis and/or EVT), as well as members of the intensive care unit such as a critical care trained nurse specialist more experienced with placing urgent peripheral lines improves patient stability and rapid triage in preparation for transportation to the CT scanner. One study investigated the role of a stroke champion, defined as a designated inpatient nurse with expert knowledge with the goal of ensuring evidence-based stroke practices are implemented and improving metrics in in-hospital code strokes.^30^ The researchers found a trend towards more accurate stroke diagnosis and an increase in the number of patients eligible for hyperacute stroke treatments following implementation of the stroke champion role.

**Figure 1.**
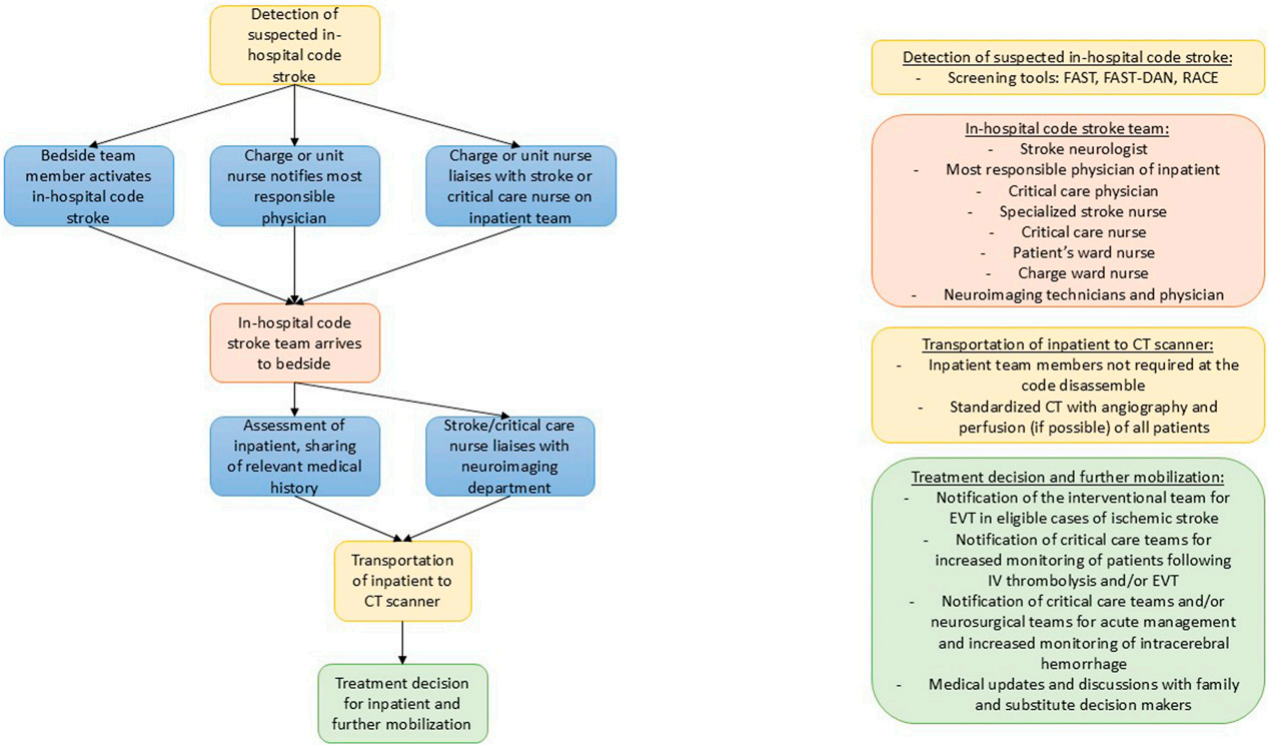
Proposed model, team members, and workflow from onset through reperfusion of the in-hospital code stroke.

Some protocols in our review, however, did not involve the on-call neurologist until after neuroimaging was completed to help make a treatment decision. This may create further delays in care if the specialists arriving at the patient’s bedside are not those that commonly assess neurologic deficits. The essence of the in-hospital code stroke response team is clinical neurosciences expertise. If there is no capacity for a specialized stroke response team such as a code stroke nurse, then members of a clinical neuroscience ward can be empowered to participate during in-hospital stroke alerts given their familiarity with acute stroke. Allied health specialists have significant insight due to the extensive amount of time they care for admitted patients, often also working on non-neuroscience wards and cross-covering. Thus, they may be some of the first individuals to recognize a change in the patient’s baseline or be in the vicinity of an in-hospital code stroke. Empowering nursing and allied health teams and leveraging their expertise is crucial for effective in-hospital code stroke responses. Empowering these professionals through education, simulation training, and the establishment of roles like the stroke champion can significantly improve stroke recognition, triage, and patient outcomes. This approach not only enhances the immediate response, but also elevates the entire team’s neuroscience expertise. One additional benefit, team-wide, is that by broad stakeholder engagement, the entire team elevates together in their neuroscience expertise. We recognize that the included members of the in-hospital stroke teams may be impacted by hospital and personnel budgets, and access to resources. Figure 1 depicts our proposed model for consideration for the in-hospital code stroke.

### Assembly and Barriers to the ad Hoc Team

A study by Nolan et al. (2003) noted that the naming of an in-hospital acute stroke was crucial to mobilize the correct team members and preparation of resources.^31^ The researchers noted that “code stroke” is typically used when the primary etiology is felt to be a stroke, with mobilization of the teams and resources within the emergency department pending arrival of an out-of-hospital patient. They further felt that families hearing “code stroke” and their loved one’s hospital room may cause undue distress if the etiology was ultimately not due to stroke. As such, they developed the term “code gray” for in-hospital acute stroke. While the premise behind the terminology of a possible in-hospital acute stroke is important, most institutions have already attributed the color system, including code gray, to mean other emergencies. Many institutions have modified this model to include a mass alert of the inpatient code stroke via pager and/or phone systems worn by the response team members. In smaller centers without in-house neurology services or less resources, involvement of the stroke unit by phone, leveraging of telemedicine and regional protocols can provide additional support in triaging cases and ensuring appropriate stroke care.

When the response team converges with the patient’s primary care specialists, challenges may arise relating to many experts assuming the care of one individual. One way to prevent interprofessional challenges may be to have a joint assessment and discussion by the stroke neurologist, intensivist, and patient’s most responsible physician to ascertain the next best steps and ensure patient stability. In our literature review, details on the neurologic examination were inconsistent and whether the NIHSS was utilized in these scenarios is unknown. Joint assessment of the patient in these cases will ensure accurate neurologic and medical examination is obtained prior to patient transport. In cases where a patient is medically unstable, whether a result of a suspected intracerebral vascular event or due to other comorbidities necessitating hospital admission, the inclusion of critical care trained physicians and nurses, and involvement of the admitted patient’s most responsible physician in the assembled team is crucial and takes precedence over the presumed stroke. The assembly and convergence of the stroke response team will further deliver potentially necessary equipment (i.e., cart with critical care equipment including monitors, cardiac defibrillator and medications) that may not be readily available on the of the patient should clinical deterioration occur. In most centers intravenous thrombolysis is often kept in the emergency department, and in the setting of an in-hospital code stroke activation, a team member can retrieve the medication while the patient is in the CT scanner, where thrombolysis can additionally be administered. Coordination with pharmacy and nursing is essential to ensure timely preparation, delivery, and administration of the medication. Once ready to mobilize the inpatient, direct communication with the imaging department will not only reduce assessment-to-imaging times, but also increase the delivery of critical news to the treating team resulting in quicker activation of treatment protocols and improve patient outcomes. Imaging protocols for in-hospital stroke activations should prioritize rapid access to neuroimaging, guided by the stroke team in collaboration with radiology. Decisions regarding imaging modalities, such as CT or MRI, are typically made by the neurologist or stroke physician on-call.

### The Era of Intervention and Use of Telemedicine

EVT has been incorporated in the standard of care for acute stroke for several years. In the seven studies comparing in- to out-of-hospital acute stroke, the incidence of patients receiving EVT with or without intravenous thrombolysis was not consistently reported. Sangha et al. (2018) found a greater number of patients receiving thrombolysis and/or EVT following implementation of their in-hospital stroke paradigm, with faster stroke recognition to groin puncture time post-intervention (median 135 minutes vs 67 minutes, *P* = 0.006).^28^ With EVT being part of the standard of care, it is important that in-hospital code stroke pathways account for access to this life-saving treatment, which includes emergent transfer of patients to comprehensive stroke centers for access to EVT.

The advent and development of Telestroke^32^ via telephone and/or video allows patients at peripheral institutions to access timely assessment and recommendations by stroke physicians at comprehensive stroke centers, and leads to not only improved functional outcomes, but improved timing and access for thrombolysis and/or EVT.^33-39^ In fact, studies have demonstrated both similar functional outcomes and mortality between patients managed at stroke centers and community hospitals,^40^ similar mortality rates and complications between patients treated with intravenous thrombolysis at peripheral sites vs at a stroke center,^41^ and a reduced need to transfer patients to a stroke center to receive appropriate care due to Telestroke use.^35^ In times of global emergency, such as during the coronavirus disease 2019 (COVID-19) pandemic, Telestroke further provides a safe and effective approach to in-hospital stroke care.^42^ Finally, Telestroke additionally provides support for rural health centers or those without in-house neurology coverage and facilitates transfer of patients for hyperacute treatments not available at some centers, making it an important potential member in the in-hospital stroke paradigm.

### In-Hospital Code Stroke and Intracerebral Hemorrhage

Early detection of an acute neurological change in the inpatient additionally holds implications for management and outcomes in hemorrhagic stroke. While less common, hemorrhagic stroke is associated with greater morbidity and mortality compared to ischemic stroke.^43^ Limiting the expansion of the hematoma and improving functional outcomes is time-dependent.^44-47^ Strategies for acute management of intracerebral hemorrhage are focused primarily on aggressive blood pressure control, cessation and reversal of therapeutic anticoagulation where applicable, and surgical intervention including extraventricular drains or decompression when there is significant risk of edema and herniation. A robust protocol for both identifying and running an in-hospital code stroke has implications to improve outcomes in patients presenting with hemorrhagic stroke.

### In-Hospital Stroke and the Road ahead

**Figure 2.**
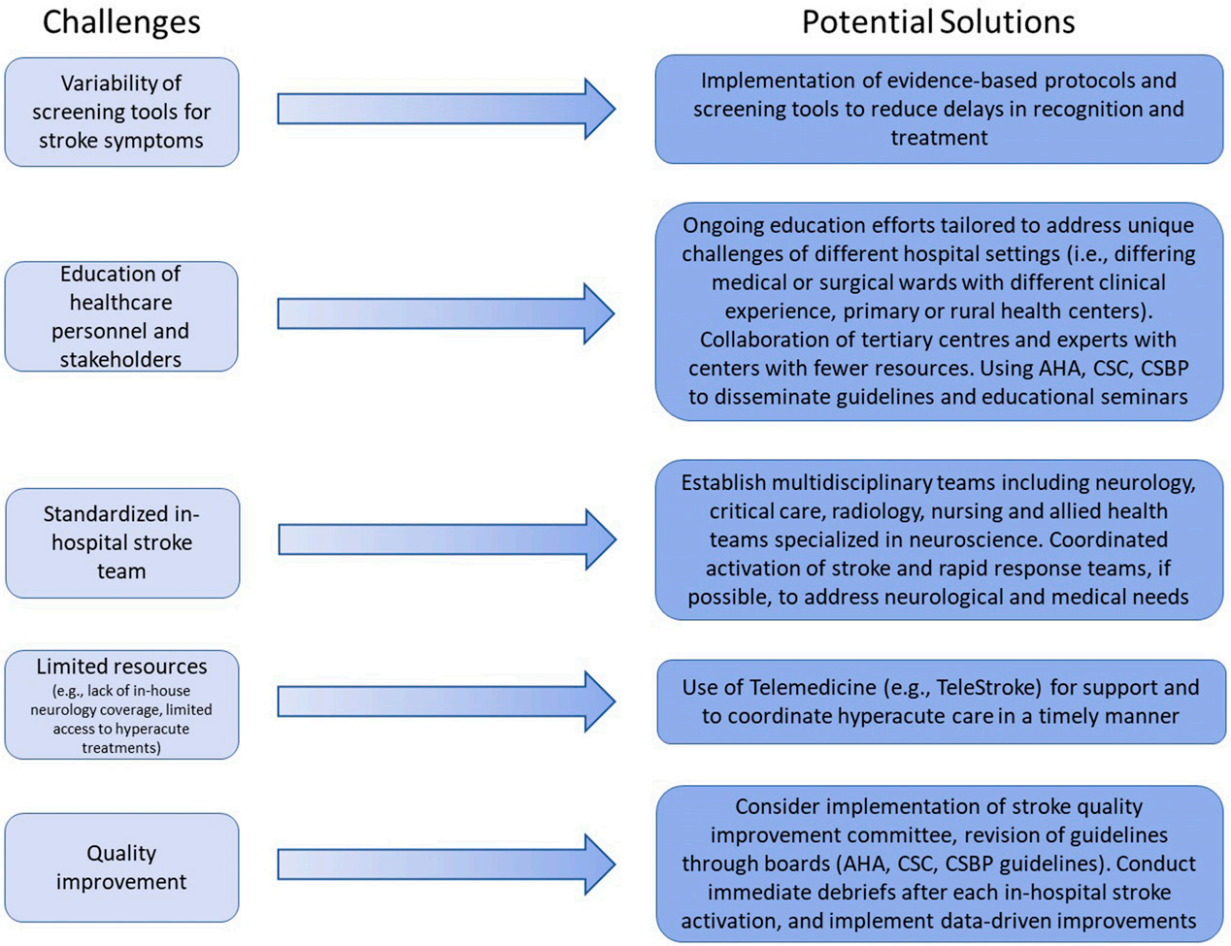
Challenges and potential solutions to the in-hospital code stroke paradigm. Abbreviations: American Heart Association (AHA), Canadian Stroke Consortium (CSC), Canadian Stroke Best Practice (CSBP) guidelines.

To improve the recognition, management, and outcomes of inpatients experiencing acute stroke, we recommend the following strategies (Figure 2):

1. Standardized inpatient stroke teams: Establish multidisciplinary teams tailored to hospital capabilities, including neurology, critical care, radiology, pharmacy, and nursing and/or allied health staff specialized in neuroscience. Empowering nursing and allied health expertise is vital for early recognition and rapid response.
2. Simultaneous activation of stroke and rapid response teams: Implement protocols that activate both teams to address both neurological and cardiopulmonary needs promptly, ensuring comprehensive patient care. The activation of both teams will additionally ensure that necessary equipment (i.e., cart with defibrillator, cardiopulmonary medications, line and intubation supplies, and intravenous thrombolysis) is readily available.
3. Standardized protocols and screening tools: Develop and implement evidence-based protocols that can be adapted to various hospital environments, facilitating rapid diagnosis and treatment initiation. Information on activating an in-hospital code stroke should be made readily available on all units. To improve the recognition of stroke, several studies have clarified the definitions of stroke symptoms to include: (1) unilateral weakness, (2) facial droop, (3) changes to speech (i.e., dysarthria and/or aphasia), and (4) altered levels of consciousness concerning for stroke even if missing other possible stroke symptoms. Clear time goals for recognition and treatment, and standardized tools (eg, checklists, order sets) may improve documentation and adherence to protocols. These resources can be disseminated via national and international boards including the American Heart and Stroke Association, Canadian Stroke Consortium, and European Stroke Organization among others.
4. Education and training: Provide ongoing education to all stakeholders with educational efforts tailored to address the unique challenges of different hospital settings. Educational efforts may include didactic and simulation-based training to improve stroke knowledge, and in-hospital stroke triage and response especially in high-risk patient populations and hospital settings. One study previously found that these efforts improved scores on a knowledge survey and increased self-assuredness to assume the role as a stroke nurse responder.^48^ Simulation training should be considered on a routine scheduled basis, including initial training of team members for in-hospital code strokes, and inclusion in educational retreats, examinations, and formal academic teaching sessions to ensure these skills remain sharp.
5. Debriefing and continuous quality improvement: Conduct immediate debriefs after each in-hospital stroke activation. There is evidence that debriefing after adverse patient safety events improves physician resilience, recovery after negative experiences, and patient care.^49^ Including debriefs following each in-hospital, and even out-of-hospital, acute stroke whether by email or immediately following patient stabilization and treatment may further improve protocols, the care provided, and neurologic outcomes. Programs should further consider the appointment of a stroke quality improvement committee consisting of key stakeholders (i.e., stroke neurology, critical care, emergency care, nursing and the allied health teams) to identify and address adherence to protocols and the subsequent outcomes. Finally, the reporting of all in-hospital stroke activations to a registry may provide further monitoring of the true incidence of in-hospital stroke and fuel future research and quality improvement measures.
6. Utilization of telemedicine: Leverage telemedicine to extend stroke expertise to hospitals lacking in-house neurology services, improving access to specialist care and advanced interventions like EVT.

## Conclusions

Despite some studies noting increased recognition of acute and mild stroke, the true incidence of in-hospital stroke may be underestimated. The inpatient has elevated risk of symptoms being under-recognized or not having access to in-hospital code stroke activation including rapid and all-hours access to emergent vascular imaging, hyperacute treatments, or in-house neurology coverage. In the present review, we have proposed a recipe for improving the recognition and management of the in-hospital stroke alert in hopes of improving functional outcomes for in-hospital stroke survivors. Future studies are required to implement these strategies and evaluate outcomes.^50-52^

## Supplemental Material

Supplemental Material - The In-Hospital Code Stroke: A Look Back and the Road Ahead Supplemental Material for The In-Hospital Code Stroke: A Look Back and the Road Ahead by Andrea M Kuczynski, W David Freeman, Lesia H Mooney, Josephine F Huang, Andrew M Demchuk, and Houman Khosravani in The Neurohospitalist.
