# Supplementary material for: The In-Hospital Code Stroke: A Look Back and the Road Ahead
Source: Neurohospitalist. 2024 Oct 29;15(2):124–32. doi: 10.1177/19418744241298035 (PMC11559452; doi:10.1177/19418744241298035)
Supplement: Supplemental Material - The In-Hospital Code Stroke: A Look Back and the Road Ahead [file sj-pdf-1-nho-10.1177_19418744241298035.pdf]

Supplementary Table 1. Characteristics and outcomes of cohort studies of pre- and post-implementation of acute in-hospital code stroke protocols.

Study	Country of Study	Number of Patients	In-hospital Code Stroke Protocol	Pertinent Outcomes
Drogemeuller et al. (2020)[29]	United States of America	N_1 = 8 (pre-implementation)	Education of nurses with an online module to improve early stroke symptom recognition and stroke activation. Written and verbal communications were given to physicians, radiologists, security personnel, pharmacists, and communications operators.	69 cases with new acute stroke, 148 stroke mimics.
		N_2 = 94 (first year post-implementation)	Utilized the FAST mnemonic: Facial drooping, Arm numbness or weakness, Slurred speech within 6 hours of last known well, T being recognition time.	Mean time to CT 18.7±7.0 minutes, with 38% achieving the target of CT ≤15 minutes from symptom onset, and 68% achieving in ≤20 minutes.

		N_3 = 123 (second year post-implementation)	Stroke team: neurologist, nurse responder, critical care physician, respiratory therapist, pharmacist, security personnel, stroke coordinator, radiology staff.	Improved completion of electronic health record flowsheet documentation (21% vs. 74% in first year post-implementation, 50% vs. 85% in second year post-implementation).
			Nurse responders were specifically trained to perform the NIHSS and respond to acute stroke.	14/63 ischemic stroke patients had IV alteplase, 7 received EVT, 4 received both.
Kassardjian et al. (2017)[7]	Canada	N_1 = 131 (pre-implementation)	Education of stroke symptoms and of code protocol to medical and surgical wards, staff including nursing staff, nursing unit managers, allied health team members.	Recognition of more milder strokes post-implementation (78.1% versus 48.1%, p=0.001).
		N_2 = 87 (post-implementation)	Laminated posters of code stroke protocol distributed to wards and residents at the hospital, pocket cards for nursing staff.	35/87 in-hospital code stroke activations were initiated post-implementation, with the remaining 52 in-hospital strokes not having activation due to unknown or >4 hours last seen

				well time.
			At symptom onset, staff contact most responsible physician who assesses the patient and either activates a code stroke (if symptom onset <4 hours from last seen well) or consults neurology and/or stroke teams.	Decreased median time from last seen well to initial assessment post-implementation (600 minutes vs. 160 minutes, p<0.01).
				Decreased median time from last seen well to neuroimaging post-implementation (925 minutes vs. 348 minutes, p<0.05).
				12 patients received IV tPA, 2 received EVT.

Kawano et al. (2021)[32]	Japan	N_1 = 42 (pre-implementation)	Workshops for hospital medical personnel including nurses and residents to improve recognition of stroke signs/symptoms, how to use the in-hospital stroke protocol, initial management of acute stroke.	Median time from stroke recognition to neurologist assessment decreased post-implementation (91 minutes vs. 35 minutes, $p<0.01$).
		N_2 = 103 (post-implementation)	Utilized the FAST-DAN mnemonic: Facial drooping, Arm numbness or weakness, Slurred speech within 6 hours of last known well, T being recognition time, Deviation of eyes, Aphasia, Neglect.	Median time from stroke recognition to neuroimaging decreased post-implementation (123 minutes vs. 74 minutes, $p=0.01$).
		N_{2A} = 54 (post-implementation, direct neurology assessment)	Informational posters with FAST-DAN and the code algorithm were put on all medical floors. Pocket guides provided to all medical personnel. Feature of new process in the hospital newsletter.	1 patient received IV tPA pre- and post-implementation. 3 patients in the pre- and 10 in the post-implementation period received EVT.

		N_{2B} = 49 (post-implementation, neurology assessment after investigations)	The patient's most responsible physician had the choice to call neurology before investigations or afterwards for patient assessment and management.	No significant differences in baseline demographics, stroke severity, and stroke symptoms between patients seen directly by a neurologist vs. after initial investigations.
				Patients seen directly by neurology vs. after investigations had reduced median time from last seen well to stroke recognition (260 minutes vs. 93 minutes, p=0.001), median time from last seen well to neurologic assessment (378 minutes vs. 145 minutes, p=0.001), and median time from stroke recognition to neurologic assessment (76 minutes vs. 16 minutes, p<0.001).

				mRS ≤2 was more common in patients directly assessed by neurology at discharge ($p<0.05$) and after 3-months ($p<0.05$) than patients assessed by neurology after investigations were completed.
Koge et al. (2017)[31]	Japan	N_1 = 25 (pre-implementation)	Education of nurses, physicians, and technologists regarding the stroke code protocol, the Cincinnati Prehospital Stroke Scale of arm drift, facial droop, or speech disorder, and management of stroke.	Reduction in median time from stroke recognition to neurology assessment (30 minutes vs. 13.5 minutes, $p<0.01$), time to first neuroimaging (50 minutes vs. 26.5 minutes, $p<0.01$), and time from neuroimaging to IV tPA (45 minutes vs. 16 minutes, $p<0.05$) post-implementation.
		N_2 = 30 (post-implementation)	The primary nurse would call in parallel a stroke unit chief nurse or on-call neurologist, emergency department nurse or the on-call patient's most responsible physician.	No difference in mRS ≤2 ($p=0.7$) at discharge pre- and post-implementation.

			Monthly conferences were held with multidisciplinary staff to discuss the protocol, past cases, and modify the protocol as needed.	11 patients received tPA, and 23 received EVT post-implementation.
Manners et al. (2019)[28]	United States of America	N_1 = 136 (pre-implementation)	Stroke education for rapid response providers including stroke symptoms via the Rapid Arterial oCclusion Evaluation (RACE) and treatment algorithms for ischemic and hemorrhagic stroke.	Reduction in stroke mimics post-implementation in patients admitted to a medicine service (71.4% vs. 33.3%, p=0.001).
				Reduction in mean time from stroke code call to neurology assessment (9.7 minutes vs. 5.1 minutes, p=0.01), stroke code call to neuroimaging (40 minutes vs. 32.4 minutes, p<0.05), and imaging to acute reperfusion therapy (45.7 minutes vs. 19.8 minutes, p=0.05) post-implementation.

			Direct communication via dedicated telephone between neurology and rapid response team. Patients were first assessed by the rapid response team and then neurology.	3 patients received IV tPA, 6 patients received EVT post-implementation.
			Graphics of the protocol were provided to all stakeholders.	No difference in rate of death (p=0.2) or discharge home (p=0.1) pre- and post-implementation.

		N_2 = 69 (post-implementation)	Rolling feedback provided to the rapid response and neurology teams.	
Yoo et al. (2016)[30]	Korea	N_1 = 28 (pre-implementation)	Regular training of nurses and physicians in the cardiology and cardiovascular surgery departments and wards on stroke recognition, screening, and the stroke code protocol.	Reduction in time from symptom onset to neurology notification (50 minutes vs. 28 minutes, p<0.05), symptom onset to neuroimaging (91 minutes vs. 41 minutes, p<0.001), and symptom recognition to notification (22 minutes vs. 9 minutes, p=0.01) post-implementation.

		N_2 = (post-implementation)	Emergency department facilities (CT) and personnel (nurses, CT technicians) were utilized as they were aware of the code stroke protocol already.	Reduction in time from symptom onset to IV tPA (120 minutes vs. 65 minutes, p<0.001), and to EVT (295 minutes vs. 165 minutes, p<0.001) post-implementation.

Abbreviations: computed tomography (CT), endovascular thrombectomy (EVT), intravenous (IV), modified Rankin scale (mRS), National Institutes of Health Stroke Scale (NIHSS), tissue-type plasminogen activator (tPA)
